# Multi-stakeholder News Recommendation Using Hypergraph Learning

**DOI:** 10.1007/978-3-030-65965-3_36

**Published:** 2020-12-09

**Authors:** Alireza Gharahighehi, Celine Vens, Konstantinos Pliakos

**Affiliations:** 5 University of Sydney, Sydney, NSW Australia; 6 Monash University, Clayton, VIC Australia; 7 University of Bari Aldo Moro, Bari, Italy; 8 University of Bari Aldo Moro, Bari, Italy; 9 University of Guelph, Guelph, ON Canada; 10 University of Caen Normandy, Caen, France; 11 University of Pisa, Pisa, Italy; 12 Norwegian University of Science and Technology, Trondheim, Norway; 13 University of Porto, Porto, Portugal; 14 UPC BarcelonaTech, Barcelona, Spain; 15 University of Porto, Porto, Portugal; 16 Fraunhofer IAIS, St. Augustin, Germany; 17 Royal Holloway University of London, Egham, UK; 18 University of Lisbon, Lisbon, Portugal; 19 University of Bari Aldo Moro, Bari, Italy; 20 University of Lisbon, Lisbon, Portugal; 21 University of Bari Aldo Moro, Bari, Italy; 22 ICAR-CNR, Rende, Italy; 23 University of Naples Federico II, Naples, Italy; 24 University of North Carolina, Charlotte, NC USA; 25 Australian National University, Canberra, ACT Australia; 26 Leibniz University Hannover, Hannover, Germany; 27 Technical University of Dortmund, Dortmund, Germany; 28 University of Southern Denmark, Odense, Denmark; 29 University of Pisa, Pisa, Italy; 30 Warsaw University of Technology, Warsaw, Poland; 31 ISTI-CNR, PISA, Italy; 32 Berlin Institute of Technology, Berlin, Germany; 33 Berlin Institute of Technology, Berlin, Germany; 34 Norwegian University of Science and Technology, Trondheim, Norway; 35 Itec, imec research group at KU Leuven, Kortrijk, Belgium; 36 Faculty of Medicine, KU Leuven, Campus KULAK, Kortrijk, Belgium

**Keywords:** News recommendation, Multi-stakeholder recommender system, Hypergraph learning

## Abstract

Recommender systems are meant to fulfil user preferences. Nevertheless, there are multiple examples where users are not the only stakeholder in a recommendation platform. For instance, in news aggregator websites apart from readers, one can consider magazines (news agencies) or authors as other stakeholders. A multi-stakeholder recommender system generates a ranked list of items taking into account the preferences of multiple stakeholders. In this study, news recommendation is handled as a hypergraph ranking task, where relations between multiple types of objects and stakeholders are modeled in a unified hypergraph. The obtained results indicate that ranking on hypergraphs can be utilized as a natural multi-stakeholder recommender system that is able to adapt recommendations based on the importance of stakeholders.

## Introduction

Classic news recommender systems try to model user preferences based on users’ previous interactions with articles. Such systems typically consist of only two types of objects, i.e. users and articles, taking into account only interactions between them [4]. Nevertheless, in many applications there are multiple types of objects and stakeholders. For example, Airbnb should take into account the preferences of both hosts and guests [1]. The same case holds in news aggregator platforms where recommender systems should take into account the preferences of their corresponding stakeholders (e.g., readers, journalists, magazines, etc.). It is therefore crucial that multi-stakeholder news recommender systems should include these different sets of objects and model the complex relations between them when generating lists of recommendations. Here, we show that the use of hypergraph ranking is a *natural* way to address multi-stakeholder news recommendation. A hypergraph is a generalization of a graph that consists of multiple node sets and hyperedges modeling high order relations between them.

There have been some studies that have applied hypergraph learning for recommendation, especially in the field of multimedia. Indicatively, in [2] the task of music recommendation was addressed as a hypergraph ranking problem, while in [7], users, images, tags, and geo-tags were modeled in a unified hypergraph model, for tag recommendation. Moreover, the authors in [6] also modeled news recommendation as a hypergraph learning problem by defining hyperedges between users, news articles, news topics and named entities. Despite its capability in modeling relations between several types of objects/stakeholders, to the best of our knowledge, hypergraph learning has not been used in the context of multi-stakeholder recommender systems.

## Methodology

Let $\mathbf {H}$ be the hypergraph incidence matrix of size $|N|\times |E|$, where *N* corresponds to nodes (users, articles, authors, topics, sources) and *E* to hyperedges. $H(n,e)=1$, if $n \in e$ and 0 otherwise. Let $\mathbf {A}=\mathbf {D}_{n}^{-1/2}\mathbf {H}\mathbf {W}\mathbf {D}_e^{-1}\mathbf {H}^{T}\mathbf {D}_{n}^{-1/2}$ be a symmetric matrix with each item $A_{ij}$ indicating the relatedness between nodes *i* and *j*. $\mathbf {D}_{n}$, $\mathbf {D}_{e}$, and $\mathbf {W}$ are the node degree, hyperedge degree, and weight matrix (here $\mathbf {W}=\mathbf {I}$), respectively. Although there are approaches to adjust or optimize the hyperedge weights (e.g. [8]), for the sake of simplicity, here we assign equal weights to all the hyperedges. We try to find a ranking vector $\mathbf {f}\in \mathbf {R}^{|N|}$ that minimizes $\varOmega (\mathbf {f})=\frac{1}{2}\mathbf {f}^{T} \mathbf {L} \mathbf {f}$, where $\mathbf {L}$ is the hypergraph Laplacian matrix and $\mathbf {R}$ represents the real numbers. This problem is extended with the $\ell _2$ regularization norm between the ranking vector $\mathbf {f}$ and the query vector $\mathbf {y} \in \mathbf {R}^{|N|}$, resulting in $Q(\mathbf {f})=\varOmega (\mathbf {f})+\vartheta ||\mathbf {f} - \mathbf {y}||_2^2$, where $\vartheta$ is a regularizing parameter. The optimal ranking vector is $\mathbf {f}^{*} = \frac{\vartheta }{1+\vartheta }\bigl (\mathbf {I} - \frac{1}{1+\vartheta } \mathbf {A} \bigr )^{-1}\mathbf {y}$ [2]. To generate the recommendation list for user $u$ in a regular recommendation task, one sets the corresponding value in the query vector to one ($\mathbf {y_{u}=1}$) and all the other values to zero.

We used a dataset from a Flemish news content aggregator website (Roularta Media Group). It consists of 3194 users, 4685 news articles and 108 authors. Each article is accompanied by title, text, tags, topics, authors and source (publisher). We defined five types of hyperedges in the unified hypergraph that are presented in Table 1. E1 connects an author to the articles he/she has written. E2 and E3 model high-order relations between the articles and their metadata. E4 represents hyperedges that connect each article to its k (we used $k=6$) most content-wise similar articles based on article embeddings. E5 models user-article interactions, connecting a user with the articles he/she has seen. To generate the article embeddings we used the CNN based deep neural network proposed by [3] based on title, text and tags of news articles.

In this dataset the majority of articles are related to a limited number of authors. This imbalanced distribution causes biased recommendations toward high frequency authors and high coverage of them in the recommendation lists. For instance, during the COVID-19 outbreak, the platform may be interested in covering more articles written by a specific author (e.g., a science journalist) or magazine in recommendation lists. This can be handled by considering a higher weight for that specific author or magazine in the query vectors. The main aim here is to demonstrate the potential and flexibility of hypergraph learning in considering different stakeholders in the model. In real cases, the platform owners should consider a trade-off between short-term utility and long-term utility. The exact weighting and strategy depend on the context and the policies of the platform owners.

**Table 1.** Hyper-edge definitions

Hyperedge	Definition
E1	Hyperedges for articles-authors
E2	Hyperedges for articles-topics
E3	Hyperedges for articles-sources
E4	Hyperedges for similar articles
E5	Hyperedges for users-articles

## Results

Three scenarios are assessed in this section and their results are shown in Table 2. The obtained results are based on per-user queries hiding/testing for each user 25 related article-interactions. Therefore only train interactions are used to form E5. The accuracy measures (nDCG@10 and Precision@10) are calculated based on test interactions. In the first scenario (baseline), the hypergraph consists of E1, E2, E3 and E5. In the second scenario, the article embeddings (E4) are added to the baseline. This way we investigate whether employing article content embeddings boosts recommendation performance. In the third scenario, we consider a higher weight for a specific author ($\alpha$) in the query vector ($\mathbf {y}$).

The obtained results show that exploiting article embeddings (E4) boosts the hypergraph ranking performance compared to the baseline. In the third scenario, adding a weight ($\beta$) for the specific author ($\alpha$) to the queries ($y_{a}=\beta$) triples the coverage of the selected author in the recommendation lists. Here, coverage is defined as the percentage of recommendation lists that contain articles of the selected author. This strategy can be used to adapt the ranked lists based on the importance of stakeholders in a specific context. Furthermore, it is vital in order to avoid bias and maximize coverage in news recommendation. The recommender can provide fairer coverage over authors by adapting their weights. It is worth mentioning that this adaptation comes at the cost of reduced precision. The weight of selected stakeholders in the query vectors can be adjusted to balance precision and coverage based on the context.

**Table 2.** Results of news recommendation

Scenarios	nDCG@10	Precision@10	Coverage
baseline (b)	0.196	0.210	0.108
(b) + article embeddings	0.202	0.220	0.116
(b) + article embeddings + multi-stakeholder	0.152	0.155	0.383

## Conclusion

In this study we showed that hypergraph ranking can naturally address multiple stakeholders in recommendations. We also showed that one can achieve better recommendation performance by adding article content embeddings. An interesting future research topic would be to dynamically adapt the queries to include more stakeholders balancing as well precision and coverage. Moreover, the trade-off between precision and coverage can be achieved by using the Pareto frontier [9]. Another interesting topic would be to diversify the recommendation lists based on the content of news articles [5]. Finally, a comprehensive evaluation study should be conducted to compare the proposed approach with other multi-stakeholder recommendation methods.
